# Genetic diversity and networks of exchange: a combined approach to assess intra-breed diversity

**DOI:** 10.1186/1297-9686-44-17

**Published:** 2012-05-23

**Authors:** Jean-François Dumasy, Christel Daniaux, Isabelle Donnay, Philippe V Baret

**Affiliations:** 1 Université catholique de Louvain, Institut des Sciences de la Vie, Embryologie moléculaire et cellulaire animale, Croix du Sud 4-5 boîte L7.07.10, 1348, Louvain-la-Neuve, Belgium; 2 Université catholique de Louvain, Earth and Life Institute, Genetics, Populations, Reproduction, Croix du Sud 2 boîte L7.05.14, 1348, Louvain-la-Neuve, Belgium

## Abstract

**Background:**

Cryopreservation of three endangered Belgian sheep breeds required to characterize their intra-breed genetic diversity. It is assumed that the genetic structure of a livestock breed depends mostly on gene flow due to exchanges between herds. To quantify this relation, molecular data and analyses of the exchanges were combined for three endangered Belgian breeds.

**Methods:**

For each breed, between 91 and 225 sheep were genotyped with 19 microsatellites. Genetic differentiations between breeds and among herds within a breed were evaluated and the genetic structure of the breeds was described using Bayesian clustering (*Structure*). Exchanges of animals between 20, 46 and 95 herds according to breed were identified via semi-directed interviews and were analyzed using the concepts of the network theory to calculate average degrees and shortest path lengths between herds. Correlation between the Reynolds’ genetic distances and the shortest path lengths between each pair of herds was assessed by a Mantel test approach.

**Results:**

Genetic differentiation between breeds was high (0.16). Overall Fst values among herds were high in each breed (0.17, 0.11 and 0.10). Use of the Bayesian approach made it possible to identify genetic groups of herds within a breed. Significant correlations between the shortest path lengths and the Reynolds’ genetic distances were found in each breed (0.87, 0.33 and 0.41), which demonstrate the influence of exchanges between herds on the genetic diversity. Correlation differences between breeds could be explained by differences in the average degree of the animal exchange networks, which is a measure of the number of exchanges per herd. The two breeds with the highest average degree showed the lowest correlation. Information from the exchange networks was used to assign individuals to the genetic groups when molecular information was incomplete or missing to identify donors for a cryobank.

**Conclusions:**

A fine-scale picture of the population genetic structure at the herd level was obtained for the three breeds. Network analysis made it possible to highlight the influence of exchanges on genetic structure and to complete or replace molecular information in establishing a conservation program.

## Background

Local livestock breeds play an important role in providing food products and environmental services and are part of the cultural heritage. In 2006, 179 of 1409, i.e. the total number of sheep breeds in the world were listed as “endangered” or “critical” and 417 other breeds had an unknown status [1]. In Belgium, ten local sheep breeds are included in the 2006 list, of which six are listed as “endangered” and four as “non endangered”. Ex situ conservation through cryobanking has been preferred to other conservation strategies (ex situ in vivo and in situ conservation) because of its lower cost and its additional benefits such as the use of cryosamples in case of an epidemic. The order of priority for the integration of the ten local Belgian sheep breeds in a cryobank has been established according to economical (population size and specific characteristics), environmental (geographical distribution and landscape management) and cultural (age, geographical origin, etc.) criteria. Three of these sheep breeds have been chosen for the conservation program: the Entre-Sambre-et-Meuse (ESM), the Mouton Laitier Belge (MLB) and the Ardennais Roux (AR) breeds.

Characterization of the intra-breed genetic diversity is a key element to select donor animals in view of ex situ conservation in a cryopreservation program. For the Belgian sheep breeds, little information is available on the pedigrees, which may result in poor evaluation of the intra-breed genetic diversity [2]. Therefore, other methods based on genetic data (microsatellites) or on information about farmers’ practices and preferences have been used. Although assessing genetic diversity between sheep breeds with microsatellite data is common practice [3-7], few studies have investigated the intra-breed genetic diversity in livestock breeds [8-10]. The observed genetic diversity in a breed can be explained by ancestral diversity, geographical isolation, natural selection, but it is mostly dependent on farmers’ practices like selection and animal exchanges. So far, the influence of such parameters has been investigated only in very specific contexts and the relationship between gene flow and genetic structure has been mainly studied at the between-breed level [11,12]. Serrano et al*.*[8] have highlighted the influence of animal exchanges in the Spanish Guadarrama goat breed, which explain the high level of subdivision observed with microsatellite analyses. In a study on the genetic diversity of the Lipizzan horse, Achman et al*.*[13] have demonstrated a strong relationship between the population structure identified with microsatellite data and the gene flow evaluated with pedigree information. In a goat population of the Vietnamese province of Ha Giang, Berthouly et al*.*[9] have measured the connectivity between farmers using least-cost path analysis in which distances between populations are expressed by differences in terms of altitude, ethnic group frequencies and probability of animal exchanges by farmers. A significant positive correlation between genetic distances and least-cost path distances was highlighted, indicating that the genetic structure is influenced by the farmer’s connectivity. Taking into account the farmer’s connectivity seems to be a relevant approach to understand the genetic structure of a population.

Tools such as network techniques can be useful to describe exchange practices. Network analysis has been used in veterinary epidemiology studies to analyze the impact of animal movements between herds on how diseases spread through a population [14,15]. In wild species, network techniques have been used to depict the interactions between individuals [16], which have been further combined with molecular methods [17]. More recently, McDonald [18] has used both network metrics and molecular methods to investigate how social interactions are related to the genetic pattern of a population of manakin birds in Costa Rica. The correlation between the degree of separation between individuals, measured by the shortest paths between pairs of individuals in a social network of 156 manakins, and their relatedness coefficient was evaluated. To our knowledge, network techniques have not been applied to study the genetic diversity of livestock.

In this study, we have investigated the intra-breed diversity at the herd level by combining two approaches: genetic markers (microsatellites) and animal exchanges between farmers. For each breed, we have correlated the genetic differentiation between herds and the network of animal exchanges between herds. Thus, this study is aimed at: (i) determining the finest genetic structure of each breed by identifying genetic groups within breeds; (ii) determining whether the network of animal exchanges between herds is linked to genetic differentiation. The results are then used to identify which individuals should be sampled to provide a good representation of the genetic diversity in the cryobank.

## Methods

### Animals

The Entre-Sambre-et-Meuse (ESM) and the Ardennais Roux (AR) sheep breeds are both bred for meat production and for the management of natural reserves. The Mouton Laitier Belge (MLB) is bred for milk production. The lower number of rams available in the MLB breed and the absence of any selection program have led some breeders to carry out outcross breeding with other breeds such as Zealand or Dutch and German Friesian breeds. For each of the three studied breeds, a first list of breeders was provided by the breeder’s associations. Other breeders were identified during farm investigations. Five hundred and fourteen sheep belonging to 53 herds and born in 84 different herds (sampled herds in Table 1) were included in the study. They were between 4 months and 11 years old. Not all the herds could be sampled but selection of the herds sampled was done to ensure a good representation of the breed’s diversity. A combination of the following criteria was used: number of animals, number of animal exchanges with other herds, historical importance and geographical position of the herd. The number of sampled animals within a herd ranged from 2 to 15 with an average of 9. In each of the 53 sampled herds, animals with different origins were chosen. Care was taken not to sample related animals (no full sibs for example) and to favor animals born in different herds according to available pedigree data. Most of the adult rams of the chosen herds were sampled. In order to identify possible crossbreeding in the MLB herds, reference samples were taken from Zealand, Dutch and German Friesian sheep. For the three breeds, samples were taken in “source herds”, which according to information collected during interviews of the breeders and specialists of the history of these breeds, have strongly contributed to the expansion and/or preservation of the pure breed. All the “source herds”, except one, exist since more than 25 years. The number of sampled “source herds” for ESM, MLB and AR breeds is 3, 3 and 2, respectively. Experimental procedures in animals were performed in accordance to the guidelines of the animal ethics committee of the Université catholique de Louvain.

**Table 1:** Number of herds and individuals and Wright’s Fst (± standard deviation) for each breed

**Breed**	**Herds of the flock book**	**Herds with identified exchanges**	**Surveyed herds**	**Sampled herds (source herds)**	**Sampled herds with at least 5 individuals**	**Adults in the surveyed herds**	**Sampled individuals**	**Fst (mean ± sd)**
**ESM**	18	20	18	12 (3)	8	604	91	0.17 ± 0.01
**MLB**	51	46	42	24 (3)	17	1176	173	0.11 ± 0.00
**AR**	205	95	58	44 (2)	17	3434	225	0.10 ± 0.01
**Reference samples**^**a**^								
Zealand	-	-	1	1	-	-	3	-
German Friesian	-	-	2	2	1	-	11	-
Dutch Friesian	-	-	1	1	1	-	11	-

^a^Reference samples: samples from breeds crossbred with the MLB breed

### Microsatellite analysis

Blood samples were collected and DNA was extracted. Individuals were genotyped with 19 microsatellite markers (see Additional file 1) from a panel recommended by the FAO [19]. DNA extraction, microsatellite amplification by Polymerase Chain Reaction (PCR) and genotyping were performed by the laboratory LABOGENA (Jouy-en-Josas, France), using a capillary sequencer (3730 DNA Analyzer; Applied Biosystems, California, USA). Information about primer sequences, allele ranges and multiplex are available from the FAO web site [19].

### Analysis of molecular data

For the three breeds and for each marker, number of alleles, observed and expected heterozygosity and Fis index were estimated using *Genetix* version 4.05.2 software [20]. *Genepop* version 3.4 [21] was used to perform exact tests for deviation from Hardy-Weinberg equilibrium (HWE) [22] for each locus, using the Markov chain Monte Carlo simulation (100 batches, 5000 iterations per batch, a dememorization number of 10 000). Unbiased estimates of the exact probabilities (P-values) were computed, and the multiple-test significance was corrected using the Bonferoni procedure [23]. *Micro-checker* software [24] was used to identify the presence of null alleles. For each breed, allelic richness was calculated using *Fstat* software version 2.9.3 [25]. Global genetic differentiation was calculated by Wright’s F-statistic Fst, evaluated with *Genetix* version 4.05.2 software [20] among the three breeds and over herds in which the sampled animals were born for each breed. Estimations of standard deviation of Fst were obtained by jack-knifing over the loci.

**Figure 1:**
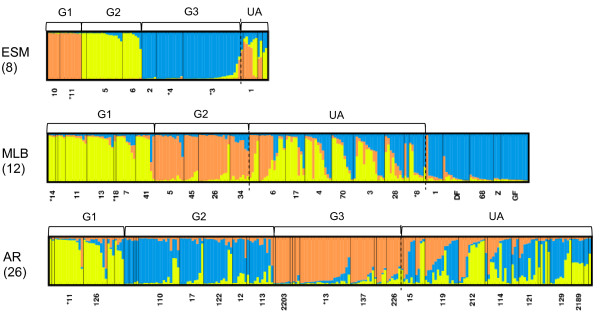
**Genetic structure of the ESM, MLB and AR populations for K = 3.** Each color represents a cluster; numbers in brackets: number of assigned herds in the genetic groups; numbers below the figures: herds with at least five sampled animals and source herds (*); G1, G2 and G3: genetic groups; GF: German Friesian; DF: Dutch Friesian; Z: Zealand; UA: unassigned individuals.

The genetic structure of each breed was investigated using a clustering method based on a Bayesian approach implemented in the *Structure* software [26], with the admixture and correlated allele frequency model [27]. In each breed, the genetic structure was studied for number of hypothetical clusters from one to ten (K = 1–10), with 10 runs for each K value with 10^5^ iterations following a burn-in period of 10^5^. No prior information about the origin of the animals was taken into account for this analysis. Membership coefficient *q* of the individual’s genomes to each hypothetical cluster and averaged $\bar{q}$ for each herd and each cluster were estimated. The most probable cluster number was identified using the method proposed by Evanno et al*.*[28]. The herds were classified into genetic groups. All the herds with a membership coefficient $\bar{q}\geq 0.7$ to the same hypothetical cluster were assigned to the same genetic group. If none of the $\bar{q}$ values were higher than 0.7, the herd was unassigned. Graphical representation of the *Structure* results was done with the *Distruct* software [29]. In Figure 1, representing the genetic structure of the breeds, herds separated by black vertical lines, are classified into their genetic group according to the decreasing value of the higher$\bar{q}$.

For each group of each breed, allelic richness, observed and expected heterozygosity, Fis indexes and exact tests for deviation from Hardy-Weinberg equilibrium (HWE) were calculated with the same software and methods as mentioned above. In addition Fst over genetic groups were evaluated for each breed.

Finally, the Reynolds’ genetic distances *D*_*r*_[30] between each pair of herds with at least five genotyped animals were computed with the *Genetix* version 4.05.2 software [20] for each breed. This measure of genetic distances is the most appropriate in this study because this distance is directly linked to the drift effect on the population structure, which is the main process shaping the structure of populations with short divergence times as in this study [31].

### Network analysis

Investigations were carried out on the breeders for each breed. We identified animal exchanges between 20, 46 and 95 herds, respectively for the ESM, MLB and AR populations. For each breed, an adjacency matrix was constructed in which for each pair of herds *i* and *j*, the *ij*^th^ entry of the matrix is 1 if there are one or more animal exchanges between them and 0 if there are none. From this matrix, a visual representation of the network can be obtained, where herds are represented as vertices and the exchanges as edges. For the calculation of network metrics, the direction of the exchanges was not taken into account (undirected networks). Since genetic distances between herds depend on the animal exchanges between the herds, networks of animal exchanges for the three breeds were compared by evaluating the average degree (*AD*) of the network of each breed. The average degree measures the number of exchanges between herds relatively to the number of herds and is expressed as *AD = 2e/n* where *n* is the number of vertices and *e*, the number of edges [32]. The average degree was calculated for the network of animal exchanges of each breed with all herds with identified exchanges, the first time, and only with herds with at least five genotyped individuals, the second time.

Genetic structure was expected to be partially explained by animal exchanges between herds. To verify this assumption, a Mantel test [33] was performed to evaluate the correlation between the matrix of genetic distances and the exchanges-based matrix called “shortest path length matrix” [34]. This latter was obtained from the network of animal exchanges for each breed. The matrix was built in the following way:

evaluation of all possible pathways (succession of edges) between two herds for each pair of herds;

identification of the shortest path(s) between each pair of herds.

The value of the distance between each pair of herds in the matrix corresponds to the number of edges separating the two herds along the shortest path(s). The shortest path lengths were calculated with the *igraph* package from the *R* statistical program [35]. All the herds with at least five genotyped animals and information about exchanges were taken into account except isolated networks of herds without exchanges with other herds to avoid infinite distances.

Shortest path lengths and Reynolds’ genetic distances were calculated for each pair of herds with at least five sampled individuals, i.e. 8 ESM, 17 AR and 17 MLB herds. The Mantel tests were performed with the *ZT* software [36] to evaluate the correlation between Reynolds’ distances and shortest path lengths. The obtained P-value is based on 10^5^ permutations.

## Results

### Analysis of molecular data

#### Genetic diversity within breeds

The numbers of herds and adult individuals for the ESM and MLB breeds surveyed cover most of the populations (nine breeders out of 69 could not be contacted). Since not all the 205 breeders known for the AR breed could be contacted, interviews were restricted to 58 breeders, i.e. all breeders with more than twenty sheep registered in the flock-book (Table 1). Null alleles were suspected only for the *OarAE129* marker in the AR breed. Thus, this marker was not taken into account for the joint analysis of the three breeds (Table 1) and for the intra-breed analysis, it was used only for the MLB and ESM breeds. Observed heterozygosities were 0.52, 0.64 and 0.63 and expected heterozygosities were 0.53, 0.65 and 0.66, respectively for the ESM, MLB and AR breeds. The average number of alleles was 6.72, 7.50 and 8.39 and the allelic richness was 6.50, 6.90 and 8.09 respectively for ESM, MLB and AR.

#### Genetic differentiation among breeds and among herds within breeds

The average genetic differentiation (Fst) among the three breeds was 0.16. The overall Fst value of pair-wise comparisons among the herds was highest for the ESM population (0.17), indicating a genetic differentiation between herds higher than in the MLB (0.11) and AR (0.10) populations.

The high Fst values within each breed suggested that the level of genetic differentiation was high among herds and motivated further investigation. According to the criterion proposed by Evanno et al*.*[28], the most probable number of clusters was two for the ESM and MLB populations (see Additional file 2). Nevertheless, results with three clusters (K = 3) were preferred since they provide a finer picture of the structure of the population than with K = 2 (Figure 1). Results based on two clusters are presented in the Additional file 3 (see Additional file 3).

In the ESM breed, the G3 group comprised two source herds originating from the splitting of a single ancient herd. The last herds (represented by “UA” in Figure 1) could not be classified in any of the three identified groups.

In the MLB breed, the first group (G1) included two source herds. The next 10 MLB herds (“UA” in Figure 1) were not classified in any of the identified groups and included unassigned individuals. The last herds represented in blue in Figure 1 were not classified in any of the MLB groups but included animals from herds of Zealand (Z) and Friesian breeds (GF and DF). Two herds with sheep registered in the flock-book of the breed clustered with the Zealand and Friesian herds because in both herds, Friesian rams (GF for herd #68 and DF for herd #1) were used for reproduction. Thus, these herds were not considered as herds of the breed. Moreover two unassigned herds (#4 and #17) include crossbred MLB sheep with Zealand sheep which explains the genetic similarity of some of their genotyped sheep with Zealand sheep.

In the AR breed, according to the criterion proposed by Evanno et al*.*[28], the most probable K value was three (see Additional file 2). One source herd was classified in the G1 group, and another in the G3 group. The last 16 herds were not classified in any of the three identified groups and included the unassigned individuals.

Genetic differentiation between groups was more than two times greater for ESM (Table 2) comparatively to the two other breeds. For the ESM breed, allelic richness was lower in the G2 and G3 groups than in the G1 group. For the two other breeds, allelic richness was similar in each genetic group. No significant deviations from Hardy-Weinberg equilibrium were observed.

**Table 2:** Genetic diversity measures in each genetic group for the three breeds

**Breed**	**N**	**AR**	**Hobs**	**Hexp**	**Fis**	**HWE**	**Fst (mean ± sd)**
**ESM**							
G1	14(2)	4.5	0.63	0.62	0.02	0.93	
G2	25(3)	3.2	0.56	0.51	−0.07	1.00	
G3	41(3)	3.2	0.49	0.47	−0.03	0.37	
							0.17 ± 0.01
**MLB**							
G1	45(8)	5.5	0.62	0.61	0.01	0.08	
G2	39(4)	5.7	0.64	0.61	−0.05	0.67	
							0.07 ± 0.02
**AR**							
G1	32(6)	5.9	0.69	0.66	−0.33	0.16	
G2	62(10)	5.6	0.63	0.64	0.03	0.54	
G3	53(10)	6.3	0.64	0.68	0.06	0.72	
							0.05 ± 0.01

N: number of individuals genotyped in each group and number of herds (in brackets); AR: allelic richness; Hobs: mean observed heterozygosity; Hexp: mean expected heterozygosity; Fis: Wright F-statistic: HWE: test for deviation from Hardy-Weinberg equilibrium; Fst: Wright F-statistic ± standard deviation (sd)

### Network analysis

#### Relation between genetic distances and average degree of the networks

The mean Reynolds’ distances between herds with at least five genotyped animals were respectively 0.21, 0.12 and 0.11 for ESM, MLB and AR (Table 3). As indicated by the higher Reynolds’ distance, genetic drift was more important for ESM by comparison with the two other breeds. This is due to the smaller population size of this breed (Table 1) and the lower connectivity between herds.

**Table 3:** Reynolds’ distances and average degree of the network of each breed

**Breed**	**Number of herds**	**Reynolds’ distance**			**Average degree**
		min	mean	max	
**ESM**	8	0.04	0.21	0.55	3.00
**MLB**	17	0.02	0.12	0.26	3.29
**AR**	17	0.02	0.11	0.29	3.76

Only herds with at least five genotyped animals are considered.

The average degree gives an evaluation of the connectivity between the herds. In our case, this network metric measures how many exchanges have occurred between herds relatively to the number of herds. Exchange networks for the three breeds are presented in Additional files 4, 5 and 6 (see Additional files 4, 5, 6). All the interviewed breeders and the breeders they quoted, and not only the breeders sampled for the genetic analyses, were represented to provide a general view of the structure of the exchanges for the studied populations. The average degrees of the exchange networks between the eight ESM herds, the 17 MLB and the 17 AR herds with at least five genotyped animals were respectively 3.00, 3.29 and 3.76. Although the number of herds is smaller for the ESM breed, comparison with the two other breeds was possible because the value of the average degree of the ESM network did not change drastically with the number of herds in the network (see Additional file 7). Regardless of the number of herds in the network of exchanges (all herds with identified exchanges (n = 20) or only herds with at least five sampled animals (n = 8)), the average degree was always smaller for ESM (non significant differences). As indicated in Table 3, the average Reynolds’ distance over pairs of herds in the network of the ESM breed is higher than the average distances observed for the two other breeds for which the average degree is higher. As expected, a higher genetic distance is a consequence of a lower connectivity between herds.

#### Correlation between genetic and network’s distances

**Figure 2:**
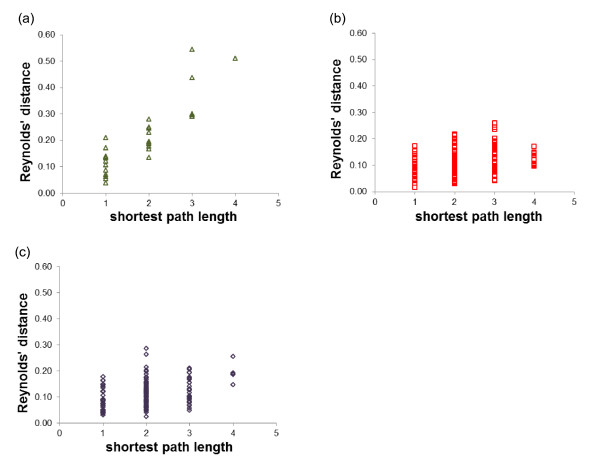
**Relation between the Reynolds’ genetic distance and the shortest path length. (a)**: ESM; **(b)**: MLB; **(c)**: AR.

It was expected that animals from herds in which breeders exchange animals would be more genetically similar than animals from herds in which no exchanges are carried out. To test this hypothesis, the correlation between genetic distances and distances based on animal exchanges was evaluated by a Mantel test. This correlation test needed two matrices of distances between each pair of herds. For each pair of herds with at least five genotyped animals, distances based on exchanges were evaluated by the shortest path length between them and Reynolds’ genetic distances were calculated. Significant correlations between these two distances were detected for the three breeds (Table 4). The observed correlations for the MLB and AR breeds were lower than those for the ESM breed. In the ESM breed, the highest genetic distances were observed between herd #10 of the G1 group and the three herds of G3. None of these three herds has had exchanges with herd #10 (Figure 2). If these three points were removed, the correlation was still higher in the ESM breed (0.83).

**Table 4:** Correlations between Reynolds’ distances and shortest path lengths evaluated by a Mantel test

**Breed**	***r***	***P***
**ESM**	0.87	0.0001***
**MLB**	0.33	0.0170**
**AR**	0.41	0.0041**

*r*: correlation coefficient; *P*: P-value calculated with 10^6^ permutations; ** significant difference at *P* < 0.05; *** significant differences at *P* < 0.001

**Figure 3:**
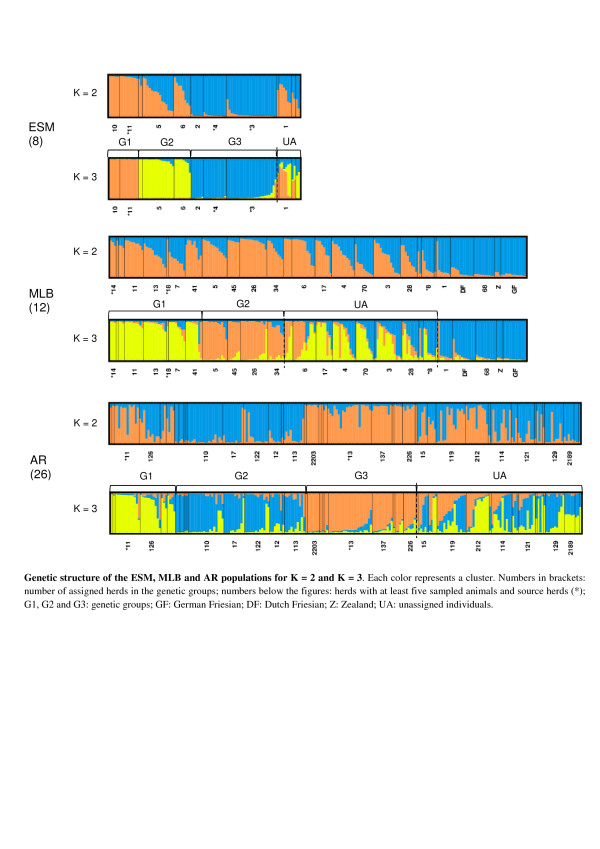
**Exchange networks and relation between Reynolds’ distance and average degree.** Exchange networks are represented for each shortest path length (SPL) value and each breed; blue vertices: herds from the complete network with the corresponding SPL value; relation between Reynolds’ distance and average degree: only the herds with at least five genotyped animals are represented; black horizontal line: median; limits of boxes: 25th and 75th percentiles; maximum limits of whiskers: 1.5 * interquartile range from the box.

**Figure 4:**
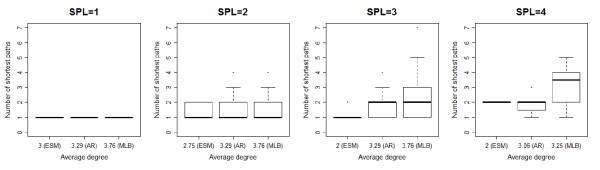
**Relation between the number of shortest paths and the average degree.** The relation is showed for each shortest path length (SPL) value; for an explanation of the graphs, cf. legend of Figure 3.

Connectivity differences assessed by the average degree of networks could explain the correlation differences between breeds. Even if two pairs of herds in two different networks have the same shortest path length, differences in genetic distances between them could be observed if the average degrees of the two networks differ. To verify this assumption, the first step consisted in calculating the average degree of networks with pairs of herds separated by the same shortest path length. Indeed the average degree depends on the ratio between the number of exchanges and the number of herds involved in these exchanges, which can vary according to the value of the shortest path length. As expected, networks with the lowest average degree (ESM) comprised pairs of herds with the highest average Reynolds’ distances (Figure 3 and Table 5). This can be explained by the smaller number of shortest paths between pairs of herds in these networks for the shortest path length values of 2 and 3 (Figure 4 and Table 5). Moreover, when the shortest path length value increased, the average degree decreased and the mean Reynolds’ genetic distance strongly increased in the ESM breed while the values of the same parameters did not vary very much in the MLB and AR breeds. This is due to a higher connectivity of the herds in these two breeds (assessed by a higher average degree) comparatively to the ESM breed, resulting in a lower genetic differentiation. This can explain the higher correlation between the Reynolds’ distances and the shortest paths lengths in the ESM breed compared with the two other breeds.

**Table 5:** Network metrics of each breed for each shortest path length

		**Shortest path length**			
		**1**	**2**	**3**	**4**
**ESM**	Mean number of shortest paths	1.00	1.30	1.20	2.00
	Mean Reynolds' genetic distance	0.11	0.21	0.37	0.51
	Number of exchanges	12	11	8	6
	Number of herds	8	8	8	6
	Average degree	3.00	2.75	2.00	2.00
**MLB**	Mean number of shortest paths	1.00	1.43	1.70	1.88
	Mean Reynolds' genetic distance	0.08	0.10	0.11	0.12
	Number of exchanges	28	28	28	26
	Number of herds	17	17	17	17
	Average degree	3.29	3.29	3.29	3.06
**AR**	Mean number of shortest paths	1.00	1.50	2.47	3.17
	Mean Reynolds' genetic distance	0.09	0.11	0.12	0.19
	Number of exchanges	32	32	32	26
	Number of herds	17	17	17	16
	Average degree	3.76	3.76	3.76	3.25

Only herds with at least five genotyped animals are considered.

In addition to the correlation analysis, a graphical method is proposed (see details in Additional file 8) to compare two types of networks: networks drawn from Reynolds’ distances information and the exchange networks drawn from information on animals’ exchanges between each pair of herds with at least five genotyped individuals.

### Characterization of donors for a cryobank

**Figure 5:**
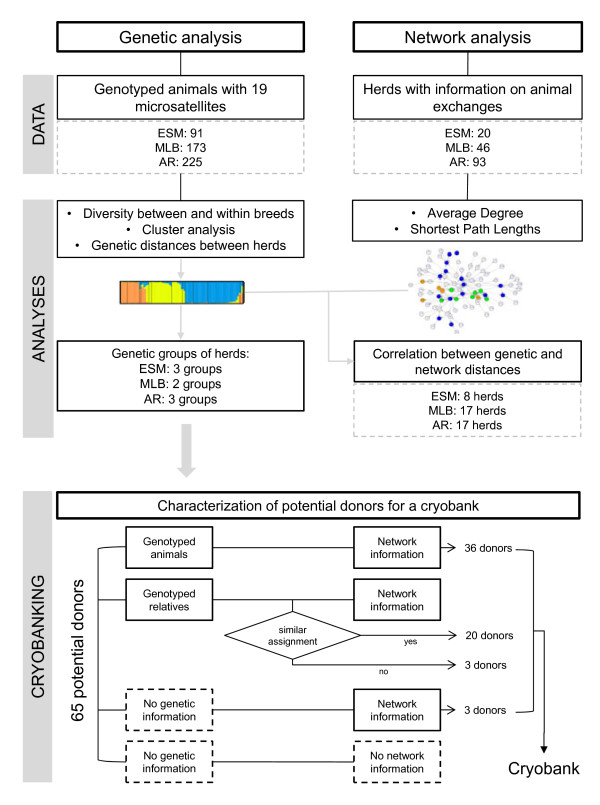
Schematic representation of the different steps from the data analysis to the constitution of the cryobank.

Since genetic and network distances were correlated, they were combined to identify herds and animals of the three breeds that could be integrated in a cryopreservation program. Fifty-eight of 65 potential donors could be genetically characterized and were classified according to a priority order for their integration in the cryobank (Figure 5). Firstly, 36 genotyped animals representative of each group in each breed were selected (32 assigned to the genetic groups and four unassigned, i.e. genotyped animals without any membership coefficient (*q*) to the hypothetical clusters higher than 0.7). Secondly, 20 non-genotyped animals with genotyped related animals and for which information on the animal exchanges from the original herd with the other herds was available were genetically characterized (17 putatively assigned to the genetic groups and 3 unassigned) using the genotypic information on their dam and sire (17) or on their grandparents (3). Thirdly, two animals were putatively assigned to the genetic groups based only on the information about networks of exchanges (see details in Additional file 9).

## Discussion

The genetic diversity and the population structure of each breed were determined by molecular analysis and significant correlations between genetic distances and distances based on animal exchanges between herds were found for each breed.

### Analysis of molecular data

The genetic diversity was studied at different levels: between the breeds (inter-breed diversity) and within the breeds (intra-breed diversity). This latter was firstly evaluated by determining the heterozygosity and the allelic richness. Secondly, the intra-breed diversity was analysed by evaluating the genetic differentiation between herds (inter-herd diversity) and between the genetic groups of herds (inter-group diversity) highlighted with Bayesian clustering in each breed.

#### Genetic diversity within breeds

The observed and expected genetic heterozygosities are smaller than the average values detected in other studies of European sheep breeds [3-6,10]. The observed smaller heterozygosity for the ESM breed (0.52), comparable to the Altamurana Italian breed (0.58) [4] and the Weisses Bergschaf Alpine breed (0.58) [5], could be the result of the smaller population size and a higher level of genetic drift. The allelic richness detected in the three breeds is similar (ESM and MLB) or higher (AR) than the average value obtained by Peter et al*.*[4] in a study on the genetic diversity of 57 European and Middle-Eastern sheep breeds (6.42). Sixteen of the 31 loci used by Peter et al*.*[4] were in common with our study.

#### Genetic differentiation among breeds and among herds

Genetic differentiation within the ESM, MLB and AR breeds, respectively 0.17, 0.11 and 0.10, was higher than those obtained by Berthouly et al*.*[9] and Serrano et al*.*[8] in their intra-breed study of the genetic diversity of goat populations, but the number of common markers is smaller (Table 6). Moreover, the intra-breed diversity is higher than the inter-breed diversity if we compare with the genetic differentiation observed between 11 Austrian sheep breeds [3], 57 European and Middle-Eastern sheep breeds [4], nine Alpine sheep breeds [5], five Italian sheep breeds [6] and five Spanish sheep breeds [10]. This high differentiation, particularly for the ESM breed, could be explained by a strong founder effect, genetic drift and differences in the choice of individuals made by breeders.

**Table 6:** Fst values observed in our study and in other studies

**Differentiation level**	**Study**	**Country**	**Species**	**Populations**	**Common markers with our study**	**Fst**
**Intra-breed differentiation (between herds)**	Our study	Belgium	Sheep			
	ESM			12 herds	-	0.17
	MLB			24 herds	-	0.11
	AR			44 herds	-	0.10
	Berthouly et al. [9]	Vietnam	Goat	10 districts	4	0.08
	Serrano et al. [8]	Spain	Goat	20 herds	2	0.07
**Intra-breed differentiation (between genetic groups)**	Our study	Belgium	Sheep			
	ESM			3 groups	-	0.17
	MLB			2 groups	-	0.07
	AR			3 groups	-	0.05
	Guastella et al. [34]	Italy (Sicily)	Pig	9 groups	0	0.12
**Inter-breed differentiation**	Our study	Belgium	Sheep	3 breeds	-	0.16
	Baumung et al. [3]	Austria	Sheep	11 breeds	7	0.08
	Peter et al. [4]	Europe and Middle-East	Sheep	57 breeds	16	0.06
	Dalvit et al. [5]	Alps	Sheep	9 breeds	7	0.06
	Bozzi et al. [6]	Italy	Sheep	5 breeds	9	0.05
	Calvo et al. [10]	Spain	Sheep	5 breeds	11	0.10

This high differentiation allowed us to identify genetic groups of herds with similar sheep in each studied breed using clustering methods. Fst values between genetic groups of the MLB and AR breeds are smaller than Fst values between herds, indicating that intra-group variation is higher than intra-herd variation. In the ESM breed, intra-herd variation is higher than intra-group variation. In comparison with the value of 0.12 observed by Guastella et al*.*[37] among nine clusters identified in the Nero Siciliano pig population, differentiation between groups is higher for the ESM breed and smaller for the MLB and AR breeds.

Information from the breeders allowed us to explain the observed substructure. Indeed, the genetic homogeneity between herds of the same group can be related to a common origin of the animals or to exchanges between herds. Moreover, suspected events of crossbreeding were confirmed for the MLB breed in which crossbred animals belong to unassigned herds or herds classified in a single group with the Friesian and Zealand sheep.

### Network analysis

#### Relation between genetic distances and average degree of the networks

The lower connectivity assessed by the smaller average degree detected in the ESM breed indicates that on average an ESM breeder exchanges animals with fewer breeders than the MLB and AR breeders. This implies a lower gene flow between herds in the ESM breed, which can explain the higher average Reynolds’ distance between herds and a higher inbreeding. For a comparison, Ortiz et al*.*[14] observed an average degree of 2.44 in the network of movements of sheep between 653 holders during the initial phases of the foot and mouth disease in the UK, which is lower than what we observed, i.e. 3.00, 3.29 and 3.76 respectively for the ESM, MLB and AR breeds. The number of exchanged animals between each pair of herds could not be taken into consideration because this information was not available for each herd. Ortiz et al*.*[14] did not take into account the number of exchanged animals. Nevertheless, this information is undoubtedly an important factor to consider when trying to explain genetic differentiation. Despite their interest, the indicators which we used are insufficient to quantitatively determine the gene flow between herds since the information is heterogeneous and refers to an appraisal of the immediate status of exchanges. Moreover, a gene flow approach requires a weighting of the exchanges in terms of animal numbers.

#### Correlation between genetic and network distances

The impact of farmers’ practices and more specifically of the animal exchanges on the genetic differentiation was confirmed by the significant correlations observed between genetic distances and distances based on the animal exchanges between herds, for the three breeds. This is in accordance with the study of Berthouly et al*.*[9]. The higher correlation between Reynolds’ distances and shortest path lengths between each pair of herds detected for the ESM breed compared with the MLB and AR breeds cannot be explained by missing information on herds and exchanges because we had access to all the available information about exchanges for the herds with genotyped animals. The smaller average degree (*AD*) observed for the ESM breed could explain the higher correlation. A higher *AD* means that more exchanges occurred between herds, implying more connectivity between them. Thus, two herds in a network with a higher *AD* are in general linked by a bigger number of shortest paths between them than in a network with a lower *AD*, for the same value of the shortest path length for the two networks. This can explain the lower Reynolds’ distances observed in the AR and MLB breeds in comparison with the ESM breed, for the networks of herds with a shortest path length value of 3 and 4. The lower increase of Reynolds’ distances in relation with the shortest path length for the MLB and AR breeds is due to a higher connectivity between herds (higher *AD*) in these two breeds. This could explain the higher correlation between Reynolds’ distances and shortest paths lengths observed in ESM.

### Characterization of donors for a cryobank

Our results show that information about animal exchanges can be used in combination with molecular data. These two types of information were used to characterize and identify herds and animals of the three Belgian sheep breeds to be integrated in a cryopreservation program. Firstly, this fine-scale study of the intra-breed diversity at the herd level allowed us to identify genetic groups and to select genotyped animals, representative of each group in each breed. Secondly, when molecular information was partial (only for relatives) or missing, information about animal exchanges was useful to assign the donor to the identified genetic groups using cluster analysis (presumed assignment). The relevance of molecular data to guide the choice of donors is higher than the relevance of network data because the network information is determined at the herd level only, is based on interviews and thus is heterogeneous in quality and depicts exchanges for a shorter span of time than the molecular information. Nevertheless, this approach was preferred to a method based on a random choice of donors when genetic information is missing and because it is neither time nor money consuming.

## Conclusions

The use of network techniques was very useful to depict animal exchanges between herds and to evaluate their level of relationship due to animal exchanges. This was necessary to calculate the correlation between genetic distances and distances based on exchanges. Moreover, differences in connectivity of the herds (in terms of animal exchanges) between breeds in relation with the level of genetic differentiation could also be highlighted with these techniques. However, until now and to our knowledge, no other study has used the networks’ techniques in combination with the analysis of genetic diversity in livestock science. Such techniques could be applied to study the diversity of livestock breeds when other information like molecular, pedigree or phenotypical data is unavailable or not reliable. Many network descriptors are available and could be useful for genetic diversity studies [32,38]. Since the farmer’s connectivity depends on topography and social structure in addition to the exchange networks [9], such elements have to be taken into account to understand the genetic structure if important differences exist in the area of study.

Moreover, network information can be valuable when molecular information is unavailable or incomplete to establish a conservation program (in situ or ex situ). A methodology to choose donors for a cryobank that are representative of the genetic diversity of a given breed based on results from both genetic and the network analyses was developed. Such an approach could be used to establish conservation programs for endangered breeds.

## Competing interests

The authors declare that they have no competing interests.

## Authors’ contributions

CD designed the study, collected data and performed statistical analyses for the ESM breed. ID and PVB participated in the design and the coordination of the study. They contributed to data analyses, critically reviewed and helped to draft the manuscript. JFD was responsible for all the steps of the study conception, the collection, the organization and the analyses of the data, and the drafting of the manuscript. All the authors read and approved the final manuscript.

## Supplementary Material

Additional file 1 **Genetic diversity measures for each locus for the three breeds.** The file contains the number of samples, the number of alleles, the observed and the expected heterozygosity, the Fis statistic and the result of the test for deviation from Hardy-Weinberg equilibrium for each breed and each locus. Click here for file

Additional file 2 **Relation between Delta K and the number of clusters K for each breed.** The file contains the graphs representing the relation between the Delta K criterion proposed by Evanno et al*.*[25] and the K value for each breed. Delta K = mean (|L(K + 1)−2 L(K) + L(K−1)|)/standard deviation[L(K)] where L(K) is the log probability of data. Click here for file

Additional file 3 **Genetic structure of the ESM, MLB and AR populations for K = 2 and K = 3.** The file contains the graphical representation of the *Structure* results with the *Distruct* software for the three breeds for K = 2 and K = 3. Each color represents a cluster. Numbers in brackets: number of assigned herds in the genetic groups; numbers below the figures: herds with at least five sampled animals and source herds (*); G1, G2 and G3: genetic groups; GF: German Friesian; DF: Dutch Friesian; Z: Zealand; UA: unassigned individuals. Click here for file

Additional file 4 **Representation of the directed network of exchanges of ESM animals.** The file contains a representation of the directed network of animal exchanges between herds of the ESM breed. Each number represents a herd. Orange circles: herds of genetic group G1; blue circles: herds of genetic group G3; green circles: herds of genetic group G2. Click here for file

Additional file 5 **Representation of the directed network of exchanges of MLB animals.** The file contains a representation of the directed network of animal exchanges between herds of the MLB breed. Each number represents a herd. Blue circles: herds of the genetic group including animals from the Friesian and Zealand breeds; green circles: herds of genetic group G1; orange circles: herds of genetic group G2. Click here for file

Additional file 6 **Representation of the directed network of exchanges of AR animals.** The file contains a representation of the directed network of animal exchanges between herds of the AR breed. Each number represents a herd. Green circles: herds of genetic group G1; blue circles: herds of genetic group G2; orange circles: herds of genetic group G3; dotted edge: link between the small network and the other herds corresponding to the smallest Reynolds’ distance between herds of the small network and the other herds. Click here for file

Additional file 7 **Number of herds and exchanges and average degree of networks for each breed.** The file contains the number of herds and exchanges and the average degree of the networks for herds with at least five sampled animals and the networks for all herds with identified exchanges for each breed. Click here for file

Additional file 8 **Comparison of genetic and exchange networks.** The file contains a comparison between the animal exchange networks and the networks based only on Reynolds’ distances (genetic networks) for the three breeds. Click here for file

Additional file 9 **Method of characterization of donors for a cryobank.** The file contains a detailed description of the method for characterizing donors for a cryobank. Click here for file
