# Supplementary material for: Genetic diversity and networks of exchange: a combined approach to assess intra-breed diversity
Source: Genet Sel Evol. 2012 May 23;44(1):17. doi: 10.1186/1297-9686-44-17 (PMC3406966; doi:10.1186/1297-9686-44-17)
Supplement: Additional file 1 — Genetic diversity measures for each locus for the three breeds. The file contains the number of samples, the number of alleles, the observed and the expected heterozygosity, the Fis statistic and the result of the test for deviation from Hardy-Weinberg equilibrium for each breed and each locus. [file 1297-9686-44-17-S1.pdf]

Genetic diversity measures for each locus for the three breeds

		OarAE129	ILSTS011	McM527	OarFCB128	OarJMP58	OarJMP29	SR-CRSP-09	CSRD0247	MAF65	MAF209	ILSTS005	OarCP34	OarFCB193	HSC	MAF70	OarFCB304	OarFCB20	HUJ616	MAF214
**ESM**	**No. of samples**	88	80	88	87	88	88	90	84	91	91	80	87	88	91	86	87	84	80	91
	**NA**	4	5	7	6	7	8	4	9	7	7	5	6	6	9	11	6	9	5	4
	**Hobs**	0.45	0.11	0.47	0.43	0.56	0.47	0.49	0.47	0.80	0.65	0.60	0.57	0.36	0.61	0.70	0.64	0.67	0.24	0.51
	**Hexp**	0.50	0.15	0.49	0.47	0.59	0.49	0.46	0.49	0.77	0.67	0.63	0.58	0.42	0.57	0.68	0.61	0.73	0.25	0.52
	**Fis**	-0.02	0.15	-0.08	0.03	-0.09	0.00	-0.18	-0.08	-0.26	-0.09	-0.16	-0.11	0.02	-0.08	-0.09	-0.12	-0.02	-0.07	-0.17
	**HWE**			***			***											***		
**MLB**	**No. of samples**	166	167	173	172	167	169	168	171	173	172	171	172	167	173	172	172	171	172	173
	**NA**	4	6	7	6	7	10	5	9	4	10	9	5	6	8	14	7	6	13	3
	**Hobs**	0.35	0.61	0.63	0.51	0.80	0.70	0.62	0.70	0.77	0.67	0.69	0.66	0.34	0.78	0.84	0.68	0.63	0.80	0.13
	**Hexp**	0.34	0.67	0.65	0.49	0.80	0.76	0.62	0.74	0.75	0.65	0.65	0.69	0.33	0.76	0.86	0.66	0.63	0.82	0.14
	**Fis**	-0.01	0.10	0.03	-0.04	0.01	0.09	0.00	0.05	-0.03	-0.02	-0.06	-0.01	-0.03	-0.03	0.03	-0.02	0.00	0.04	0.08
	**HWE**																			
**AR**	**No. of samples**	204	225	225	226	223	222	225	225	225	224	225	224	222	224	226	224	223	225	225
	**NA**	5	6	8	7	11	12	5	8	7	9	6	6	8	10	13	8	12	10	5
	**Hobs**	0.43	0.53	0.68	0.47	0.80	0.66	0.55	0.73	0.74	0.75	0.47	0.68	0.13	0.86	0.83	0.58	0.84	0.50	0.50
	**Hexp**	0.68	0.63	0.76	0.52	0.84	0.70	0.60	0.76	0.78	0.77	0.47	0.69	0.12	0.82	0.88	0.61	0.86	0.54	0.52
	**Fis**	0.38	0.16	0.11	0.09	0.04	0.05	0.09	0.04	0.05	0.03	-0.01	0.03	-0.01	-0.05	0.06	0.05	0.03	0.08	0.03
	**HWE**	***	**	*																

NA: number of alleles; Hobs: mean observed heterozygosity; Hexp: mean expected heterozygosity; Fis: F-statistic; HWE: test for deviation from Hardy-Weinberg equilibrium (*: $P<0.05$; **: $P<0.01$; ***: $P<0.001$)
