# Supplementary material for: Genetic diversity and networks of exchange: a combined approach to assess intra-breed diversity
Source: Genet Sel Evol. 2012 May 23;44(1):17. doi: 10.1186/1297-9686-44-17 (PMC3406966; doi:10.1186/1297-9686-44-17)
Supplement: Additional file 2 — Relation between Delta K and the number of clusters K for each breed. The file contains the graphs representing the relation between the Delta K criterion proposed by Evanno et al.[25] and the K value for each breed. Delta K = mean (|L(K + 1)−2 L(K) + L(K−1)|)/standard deviation[L(K)] where L(K) is the log probability of data. [file 1297-9686-44-17-S2.pdf]

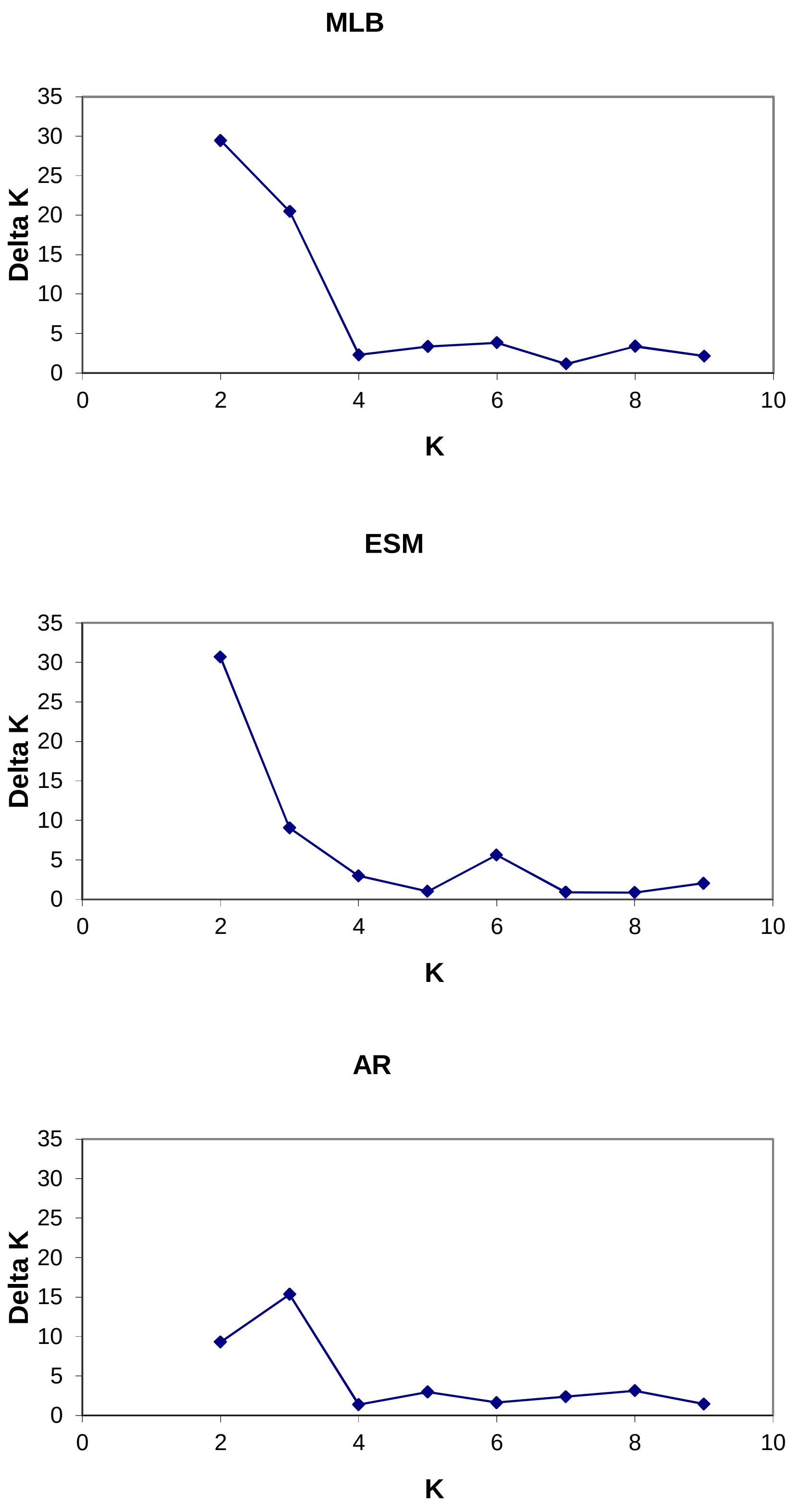

Relation between Delta K and the number of clusters K for each breed

Delta K = mean [|L(K+1)-2L(K)+L(K-1)|]/standard deviation[L(K)] where L(K) is the log probability of data.
