# Supplementary material for: Genetic diversity and networks of exchange: a combined approach to assess intra-breed diversity
Source: Genet Sel Evol. 2012 May 23;44(1):17. doi: 10.1186/1297-9686-44-17 (PMC3406966; doi:10.1186/1297-9686-44-17)
Supplement: Additional file 3 — Genetic structure of the ESM, MLB and AR populations for K = 2 and K = 3. The file contains the graphical representation of the Structure results with the Distruct software for the three breeds for K = 2 and K = 3. Each color represents a cluster. Numbers in brackets: number of assigned herds in the genetic groups; numbers below the figures: herds with at least five sampled animals and source herds (*); G1, G2 and G3: genetic groups; GF: German Friesian; DF: Dutch Friesian; Z: Zealand; UA: unassigned individuals. [file 1297-9686-44-17-S3.pdf]

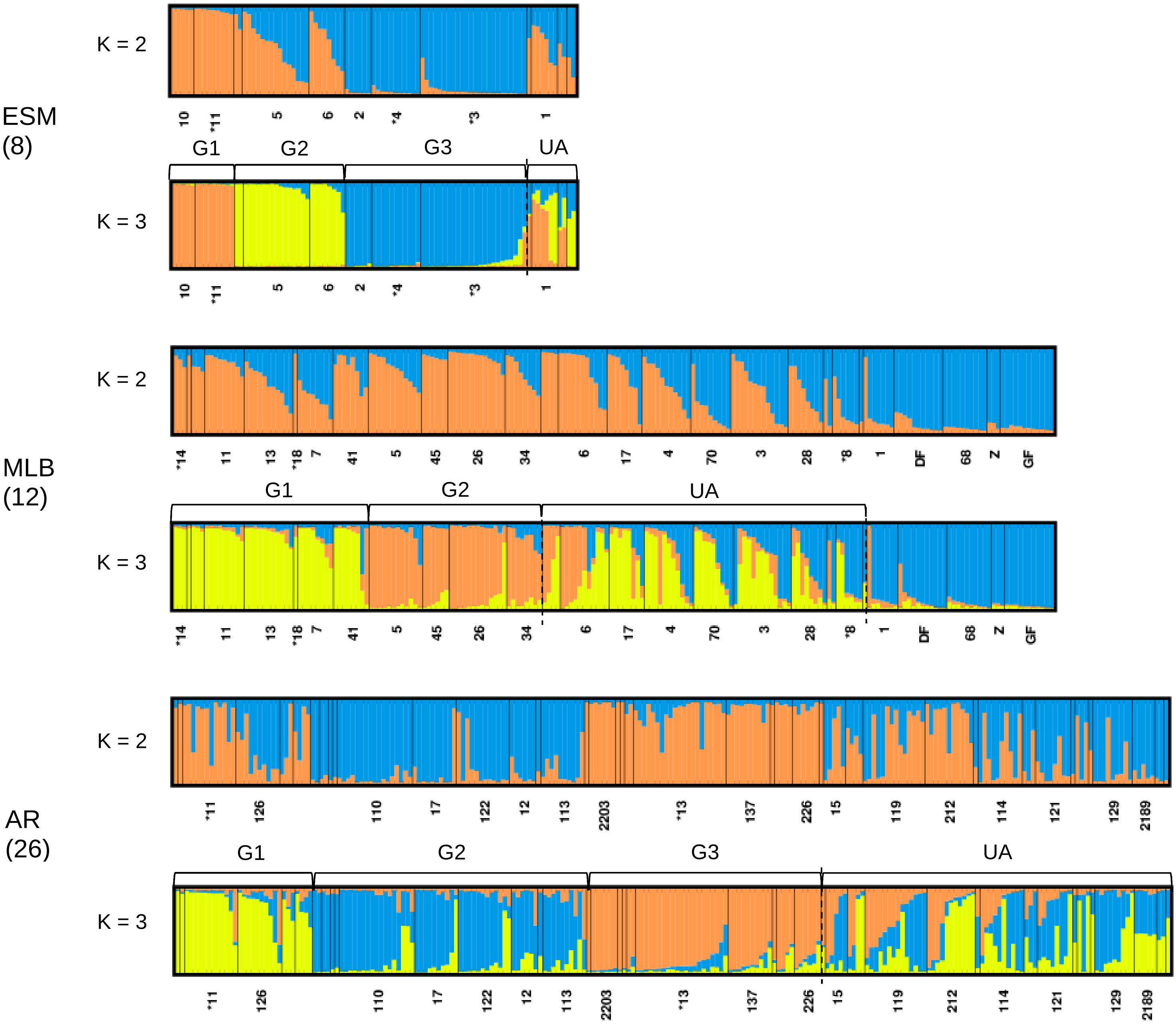

Genetic structure of the ESM, MLB and AR populations for K = 2 and K = 3. Each color represents a cluster. Numbers in brackets: number of assigned herds in the genetic groups; numbers below the figures: herds with at least five sampled animals and source herds (*); G1, G2 and G3: genetic groups; GF: German Friesian; DF: Dutch Friesian; Z: Zealand; UA: unassigned individuals.
