# Supplementary material for: Genetic diversity and networks of exchange: a combined approach to assess intra-breed diversity
Source: Genet Sel Evol. 2012 May 23;44(1):17. doi: 10.1186/1297-9686-44-17 (PMC3406966; doi:10.1186/1297-9686-44-17)
Supplement: Additional file 6 — Representation of the directed network of exchanges of AR animals. The file contains a representation of the directed network of animal exchanges between herds of the AR breed. Each number represents a herd. Green circles: herds of genetic group G1; blue circles: herds of genetic group G2; orange circles: herds of genetic group G3; dotted edge: link between the small network and the other herds corresponding to the smallest Reynolds’ distance between herds of the small network and the other herds. [file 1297-9686-44-17-S6.pdf]

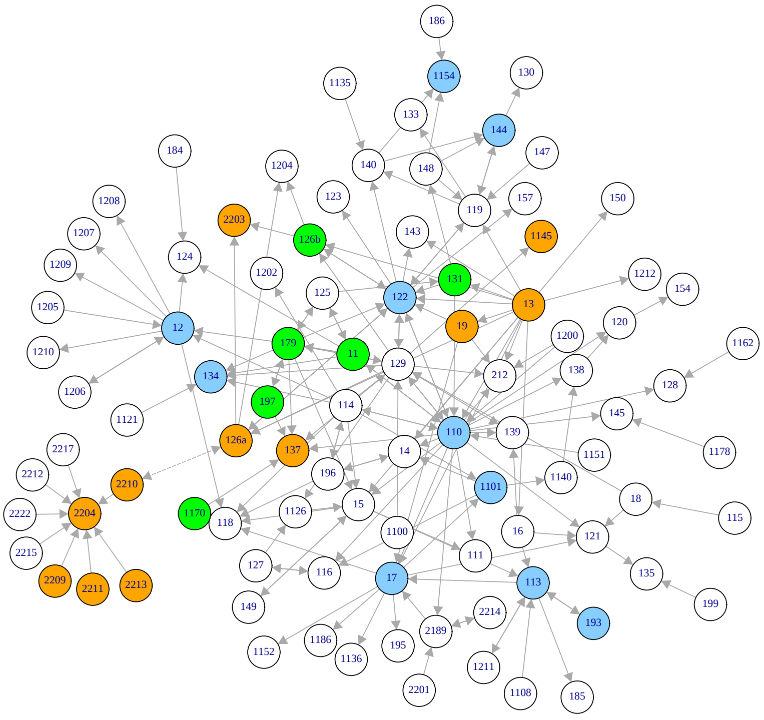

Representation of the directed network of exchanges of AR animals

Each number represents a herd. Green circles: herds of the genetic group G1; blue circles: herds of the genetic group G2; orange circles: herds of the genetic group G3; dotted edge: link between the small network and the other herds corresponding to the smallest Reynolds' distance between herds of the small network and the other herds.
