# Supplementary material for: Genetic diversity and networks of exchange: a combined approach to assess intra-breed diversity
Source: Genet Sel Evol. 2012 May 23;44(1):17. doi: 10.1186/1297-9686-44-17 (PMC3406966; doi:10.1186/1297-9686-44-17)
Supplement: Additional file 7 — Number of herds and exchanges and average degree of networks for each breed. The file contains the number of herds and exchanges and the average degree of the networks for herds with at least five sampled animals and the networks for all herds with identified exchanges for each breed. [file 1297-9686-44-17-S7.pdf]

Number of herds and exchanges and average degree of networks for each breed

Network	Number of herds	Number of exchanges	Average Degree
**ESM**	8	12	3.00
**ESM (all)**	20	31	3.10
**MLB**	17	28	3.29
**MLB (all)**	46	85	3.70
**AR**	17	32	3.76
**AR (all)**	84	137	3.26

Herds with at least five sampled animals and all herds with identified exchanges (“all”) are both considered.
