# Supplementary material for: Genetic diversity and networks of exchange: a combined approach to assess intra-breed diversity
Source: Genet Sel Evol. 2012 May 23;44(1):17. doi: 10.1186/1297-9686-44-17 (PMC3406966; doi:10.1186/1297-9686-44-17)
Supplement: Additional file 8 — Comparison of genetic and exchange networks. The file contains a comparison between the animal exchange networks and the networks based only on Reynolds’ distances (genetic networks) for the three breeds. [file 1297-9686-44-17-S8.pdf]

Comparison of genetic and exchange networks

Exchange information used for the setting up of networks is a proxy of the gene flow between herds but differs from molecular information because exchange information is based on breeders' interviews and thus is heterogeneous in quality and depicts exchanges for a shorter span of time. In addition number and frequencies of exchanges between herds were not taken into account with this information. Comparison between exchange networks and networks based only on Reynolds' distances (genetic networks) were made to verify the compatibly between the two types of information.

In the genetic networks, edges between any pair of herds with at least five genotyped individuals were drawn if the Reynolds' distance between herds was lower or equal to a threshold value which corresponds to the smallest value of the ratio (A+B)/C where A is the number of edges only present in the genetic network, B is the number of edges only present in the exchange network and C is the number of edges shared by the two compared networks. This ratio was calculated for each increment of 0.01 of the Reynolds' distance in the interval limited by the minimum and the maximum values in each breed. Threshold values and parameters A, B and C are indicated in the table below.

Values of parameters and threshold of Reynolds' distance for each breed

Breed	A	B	C	(A+B)/C	Threshold
ESM	2	1	11	0.27	0.17
MLB	22	12	16	2.13	0.08
AR	39	11	21	2.38	0.09

A: number of edges only present in the genetic network
B: number of edges only present in the exchange network
C: number of edges shared by the two compared networks

As indicated in the table, the correspondence between the two types of networks is the highest for the ESM breed in comparison with the two other breeds. Eleven of the 12 identified edges of the exchange network are also in the genetic network which includes only two new edges. The smaller similarity observed for the two other breeds comes from a higher number of

edges only present in one of the two networks and a proportionally smaller number of common edges. Differences of configuration of the two networks for the ESM breed are shown in the representation of network below.

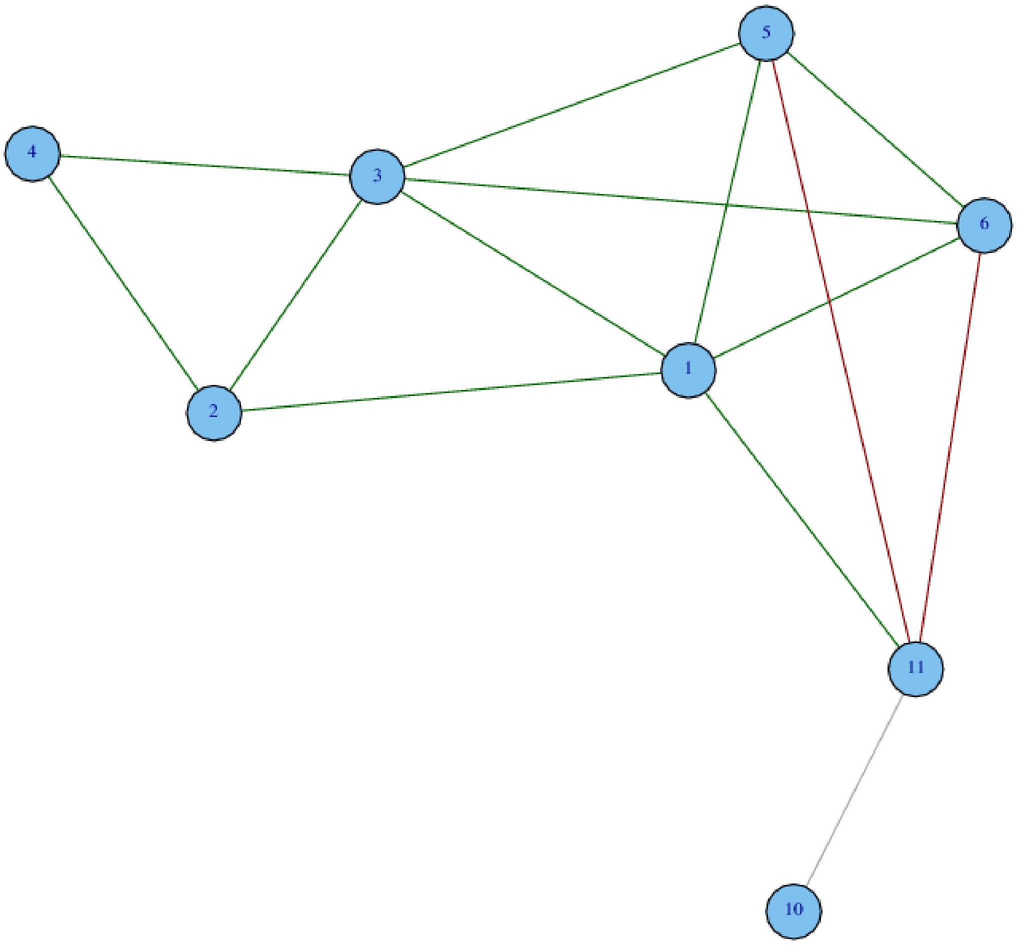

Representation of the network of ESM herds. Each number represents a herd. Green edges: edges present in both genetic and exchange networks; red edges: edges only present in the genetic network; grey edge: edge only present in the exchange network

Differences between the two networks (red and grey edges) can come from missing (red edges) or wrong (grey edge) information about exchanges or from the fact that the exchange network does not carry information about the amount and the frequency of exchanges (grey edges) and depicts direct gene flow between herds (red edges) and not indirect connections.
