# Supplementary material for: Genetic diversity and networks of exchange: a combined approach to assess intra-breed diversity
Source: Genet Sel Evol. 2012 May 23;44(1):17. doi: 10.1186/1297-9686-44-17 (PMC3406966; doi:10.1186/1297-9686-44-17)
Supplement: Additional file 9 — Method of characterization of donors for a cryobank. The file contains a detailed description of the method for characterizing donors for a cryobank. [file 1297-9686-44-17-S9.pdf]

Method of characterization of donors for a cryobank

Since genetic and network distances were correlated, the combination of these two types of information was used to identify herds and animals of the three breeds to be integrated in a cryopreservation program. Fifty-eight of the 65 potential donors could be genetically characterized and were classified according to a priority order for their integration in the cryobank (Figure 5).

- Firstly, 36 genotyped animals representative of each group in each breed were selected (32 assigned to the genetic groups and four unassigned, i.e. genotyped animals without any membership coefficient (q) to the hypothetical clusters higher than 0.7).
- Secondly, 23 non-genotyped animals with genotyped related animals and for which information on animal exchanges between the original herds and the other herds was available were identified and 20 of these could be characterized (17 putatively assigned to the genetic groups and three unassigned):
    - Fifteen of the 17 animals with genotyped mothers have a father originating from the same herd as the mother or from a herd for which animal exchanges took place with the herd of the mother or herds of the genetic group of the mother. These animals were putatively assigned to the genetic group of the mother. The fathers of the two other animals originated from herds in which exchanges with herds of different genetic groups had occurred and could not be assigned.
    - Two animals with genotyped fathers were putatively assigned to the genetic group of the father according to the same criteria.
    - Three of the four animals with one genotyped grandparent were putatively assigned to the same genetic group as those of the herd of the grandparent, since they originate from the same herd or from a herd in which exchanges occurred preferentially with the

herd of the grandparent. This is not the case for the last one which could not be assigned.

- Thirdly, three potential donors were identified for which only information on animal exchanges was available. One of the three originated from a herd without genotyped animals but in which exchanges with herds from the same genetic group occurred. This animal was putatively assigned to this group. The two other donors originated from herds with genotypic information but neither their parents nor their grand-parents were genotyped. The first comes from a herd in which exchanges occurred only with herds of the same genetic group and was thus putatively assigned to this group. This is not the case for the second animal which could not be assigned to any genetic group.
